# Parents’ experience with child safety restraint in China

**DOI:** 10.1186/1471-2458-14-318

**Published:** 2014-04-07

**Authors:** Xiaojun Chen, Jingzhen Yang, Corinne Peek-Asa, Liping Li

**Affiliations:** 1 The First Affiliated Hospital of Shantou University Medical College, Shantou, China; 2 Injury Prevention Research Center, Shantou University Medical College, Shantou, China; 3 The University of Iowa Injury Prevention Research Center, Iowa, USA; 4 Department of Social and Behavioral Sciences, Kent State University, Kent, USA; 5 Department of Occupational and Environmental Health, University of Iowa, Iowa, USA

**Keywords:** Child safety seat, Interview, Qualitative research

## Abstract

**Background:**

Child safety restraints are effective measures in protecting children from an injury while traveling in a car. However, the rate of child restraint use is extremely low in Chinese cities. Parent drivers could play an important role in promoting child safety restraint use, but not all of them take active responsibility.

**Methods:**

This study used a qualitative approach and included 14 in-depth interviews among parents with a child, under the age of 6, living in Shantou City (7 child safety restraint users and 7 non-users). Purposive sampling was used to recruit eligible parent drivers who participated in a previous observation study. Interview data were collected from March to April 2013. The audio taped and transcribed data were coded and analyzed to identify key themes.

**Results:**

Four key themes on child safety restraint emerged from the in-depth interviews with parents. These included 1) Having a child safety restraint installed in the rear seat with an adult sitting next to the restrained child is ideal, and child safety restraint is seen as an alternative when adult accompaniment is not available; 2) Having effective parental education strategies could help make a difference in child safety restraint use; 3) Inadequate promotion and parents’ poor safety awareness contribute to the low rate of child safety restraint in China; 4) Mandatory legislation on child safety restraint use could be an effective approach.

**Conclusion:**

Inadequate promotion and low awareness of safe traveling by parents were closely linked to low child safety seat usage under the circumstance of no mandatory legislation. Future intervention efforts need to focus on increasing parents’ safe travel awareness combined with CSS product promotion before the laws are enacted.

## Background

Motor vehicles crashes are one of the leading causes of death in children from birth to 14 years of age worldwide [1]. Drowning and traffic crashes are the top two causes of early childhood injury deaths in China [2,3]. Each year in China, roughly 100,000 people are killed in road traffic crashes [4]. Children, aged 1 to 20 years, account for more than 12 percent of total road traffic deaths [5]. Child passenger safety seats are designed to protect children from crash-related injuries; when correctly installed and used, they can reduce the risk of crash-related fatality by up to 71% and the risk of serious injury by up to 67% [6]. Unfortunately, child safety seat (CSS) use in China is extremely low, although motorization has increased rapidly. Recent studies show that 93.9% of children under the age of 7 in Shanghai were not restrained [7]. Currently, China’s seat belt laws do not specify the age of a passenger who is prohibited in the front seat. China also has no law requiring the use or proper installation of child safety restraints.

Despite positive attitudes about safety seats, our previous observational study conducted in the City of Shantou in 2012 found that less than 1% of the 3,333 observed cars utilized a child safety seat [8]. A critical gap was identified between positive attitudes towards child safety restraint and very low usage in our study sample, suggesting that further research is needed to explore the reasons why or why not child safety restraint was used. Thus, this study utilized a qualitative method aimed to explore parent drivers’ perceptions and experiences regarding use of child safety restraint, and to identify the differences in the perceptions and experiences between child safety restraint users and non-users. According to the Health Belief Model, individuals’ beliefs about health problems, perceived benefits of action and barriers to action, and self-efficacy explain why individuals are engaging or not engaging in health-promoting behavior [9]. In the context of this study, the decisions of parents to use or not use child safety restraint are likely influenced by their perceived risk of not using CSS (e.g., poor knowledge), perceived benefits of using CSS (e.g., protecting child from an injury), and perceived barriers of using CSS (e.g., high prices) [8]. Findings of this study could offer insights about why or why not child safety restraints are used, thus providing direction for future intervention strategies to improve such use.

## Methods

### Interview guide development

Guided by the Health Belief Model, the principles of a qualitative methodological approach, existing published literature on CSS [7,10,11], and the findings from our previous observational study, the interview guide was developed for inclusion of both parents of CSS users, and parents of CSS non-users who had resistance to using CSS. Following the review and feedback by two experts who have extensive experience in traffic safety research and instrument development, the updated interview guide was pilot tested with two parent drivers before it was finalized. In addition to demographic questions, the final interview guide was comprised of four sets of questions and probes, including: perception of child safety while traveling in a car, attitude towards CSS, facilitating factors and/or barriers associated with use or non-use, and opinions on future effective intervention strategies to promote use.

### Participants selection

The participants were selected from the previous observational study, of which 1069 drivers completed the questionnaire survey and some of them left contact information and indicated willingness to be contacted for further study. Our previous findings showed that nearly 60% of CSS users had a child under the age of 6, suggesting that families with children under 6 might be a potential group to target for promoting CSS use. Thus, purposive sampling was used to recruit parent participants. Eligible parents were those who currently lived in the downtown area of Shantou, with a child under the age of 6 living in the same household, and who had been a primary driver and driving more than one time every week for at least 2 years. A total of 20 participants who met the selection criteria were contacted conveniently to invite them to participate in the study. Four of 20 declined participation due to busy schedule, and 16 agreed to participate in the study. When parents agreed to be interviewed, they were contacted to explain the purpose and procedures of the study and to schedule an interview in a private room at a public place (e.g., tea house) with a location, date, and time that were convenient for the parents. After signing the informed consent, parents were asked questions about their perception and practices of child safety restraint. A total of 14 eligible parents were interviewed. Those who had used CSS (n = 7) were categorized as users, while those who had never used CSS (n = 7) comprised a non-user group. Open enrollment was used and continued until the parents’ responses on the perceptions and practices of CSS became similar across multiple participants. When thematic saturation was reached, enrollment stopped. The institutional review board of the Shantou University Medical College approved this study and consent process.

### Data collection

The interviews were conducted face-to-face by one of the authors who were trained in qualitative interviewing and the study protocol. Each of the interviews lasted 35 to 50 minutes. The interview began with a general question asking participants to describe how they knew about CSS, and then continued with more specific questions on their experiences of either using or not using CSS, facilitating factors and/or barriers associated with use or non-use, and their opinions for future intervention strategies. All of the interview questions used an open-ended structure, which allowed parents to speak freely. After the completion of the interview, the parents were given a small gift of child stationery and a toy in appreciation for their time. All interviews were audiotaped and transcribed verbatim into text. Our study adhered to the RATS guidelines for reporting qualitative studies.

### Data analysis

Interview transcripts were repeatedly read by the research team to identify recurring patterns and their links to the theoretical framework [12]. The interviews were recorded and transcribed verbatim. The transcripts were then imported into software NVivo 10.0 and were then coded and analyzed thematically. The coding system was based on the content of the data. Line-by-line coding of the interview transcripts was completed by two researchers, then followed by group sessions of the research team to discuss and refine codes until consensus could be achieved. Differences in independent coding were discussed among research team members until consensus was reached. After this process, key themes or recurrent and unifying ideas that described the parents’ opinions and experiences were summarized in themes and sub-themes. A set of coded transcripts was ultimately produced and categorized into final themes. Some valuable perspectives shared by the parents on general child traffic safety were also analyzed and included, although these departed from the central themes. Finally, the data and results were evaluated by the bilingual and bicultural research team.

## Results

### Characteristics of participants

Of 14 parents interviewed, 7 parents were CSS users while 7 were non-users, with ages ranging from 29 to 34 (Table 1). Two of the 14 participants were fathers and the rest were mothers. All participants had a bachelor degree or higher education, with monthly income levels at or above the city’s average. They all owned a car and drove daily to and from work. The average years of driving were 4.6 years.

**Table 1:** Profiles of respondents for in-depth interviews

**CSS User group**							
**No**	**Gender (male/female)**	**Age**	**Level of education (degreed)**	**Monthly income (Yuan)**	**Driving experience (year)**	**Child’s age (year/month)**	**Time of CSS use (year/month)**
A	F	34	Doctor	7000	4	5	1.5 y~
B	F	31	Bachelor	3000	7	9 m	0
C	F	33	Bachelor	4000	9	3	2 y~
D	F	31	Bachelor	2300	7	3	1 y
E	F	29	Master	4000	7	15 m	6 m~
F	F	34	Bachelor	6000	3	5	2 y
G	F	34	Bachelor	7000	3	6	5y~
**CSS- Non-user group**							
A	F	34	Master	5000	7	5	0
B	F	33	Master	5000	2	15 m	0
C	F	34	Bachelor	3500	2.5	4	0
D	F	34	Bachelor	4000	2	6	0
E	M	30	Bachelor	6000	3	1	0
F	M	31	Bachelor	3500	6	4	0
G	F	32	Bachelor	4500	3	3	0

Note: 1.5 y ~ stands for CSS use for one and half years and will continue to use. F for female, M for male. Mother B in CSS User Group used CSS less than one month.

### Four Key themes identified

Sub-themes under four key themes, with supported quotes, were list in Table 2. The following were the four key themes identified based on parents’ experiences and practices regarding to CSS. Under each theme, the data on the differences between CSS users and non-users were presented and compared:

**Table 2:** Sub-themes from specific topics under four key themes

	**Theme**	**Quote**
1)	**Having a child safety restraint installed in the rear seat with an adult sitting next to the restrained child is ideal, and child safety restraint is an alternative when adult accompaniment is not available.**	
	Both parents in CSS user group and non-use group indicated that infants are not suitable to sit in CSS; they need to be secured in the arm of an adult. When children are old enough to school age, CSS is no longer necessary.	I would like to have an adult, my mother or my wife, to hold my baby in arm and sit on the left side of the back seat. CSS is the second choice since it is not ready available for use. (father E in CSS non-use group)
	B. For non-user group, parents often explained that short distance and slow speed in the city would not cause serious injury even in a crash; CSS is not that important.	We don’t ask my child to wear the seat belt because most of the time we are driving in the downtown area. (mother D in CSS non-use group)
2)	**Having effective parental strategies could help make a difference in child safety restraint use.**	
	A. Parents in CSS user groups would make every effort to seat their children in the CSS as long as possible.	I tried to convince him by saying that if he sits in the CSS, he could go anywhere or get a candy. (mother D in CSS group)
	B. Parents in CSS non-user groups would persuade their children sitting in the rear. What they worry most is their children would not like to sit in the CSS if they buy it.	I heard that children don’t like to sit in the CSS, even if they do, the time they can use CSS is not long. So it is impractical. It is a waste even if I buy it.(mother B in CSS non-use group)
3)	**Inadequate promotion and parents’ poor safety awareness contribute to the low rate of child safety restraint in China.**	
	A. All of the parents reported that they have once seen the car crash video in which children were thrown out of the car. Most parents in CSS user groups indicated that using the safety restraint could possibly reduce the harm. Most parents in the other groups indicated that strictly obeying the traffic law is the way to prevent it.	No one knows it, how can we use it. I heard it and want to buy it, but I fail to persuade my husband because I don’t know much about it. (mother A in CSS non-use group)
	B. Parents in the user group focused on the quality of CSS, as bad quality of CSS would harm the child if a crash occurred.	I would like to choose a better CSS for my baby because I know from TV that a bad quality CSS might not protect the child in a crash when compared to a good one.(mother A in CSS group)
4)	**Mandatory legislation on child safety restraint use could be an effective approach.**	
	Only severe punishment could constrain people from dangerous behavior, whether they have high safety consciousness or not.	People may not act unless severe punishment is in place. A fine is needed in the current Chinese society.(mother E in CSS group)
	Law enforcement could be better carried out if in combination with public education on child safety traveling.	This is not solely the responsibility for the traffic department; educational institutions should also be involved. (father E in non-use group)

*1. Having a child safety restraint installed in the rear seat with an adult sitting next to the restrained child is ideal, and child safety restraint is seen as an alternative when adult accompaniment is not available*

Parents in CSS user group described their experiences using CSS. They all agreed that the ideal practice for a child passenger in a car is to have CSS installed in the rear seat, and have an adult sitting next to the restrained child. Non-CSS users though that a child should sit in the back seat with an adult, and did not routinely identify restraint as important.

The backseat behind the driver is the safest because the driver would respond immediately to any unsafe event to protect himself or herself. CSS is designed to protect children from crash-related injury, as a safety seat belt for adults. Ideally, having an adult sitting next to the restrained child can help ensure the safety seat remains fastened and child is comfortable with CSS, because of driving the car, I can’t see that.

— mother A in CSS group

Most parents in the CSS non-user group thought the safest situation was to have a child sit in the backseat accompanied by an adult. Only two respondents mentioned a CSS as necessary.

I would like to have an adult, my mother or my wife, to hold my baby in arm and sit on the left side of the back seat. CSS is the second choice since it is not ready available for use.

— father E in CSS non-use group

Almost all participating parents, either CSS users or non-users, agreed that the front seat was unsafe; not only the child sitting there would distract the driver, but also thought that injury cause by sitting in the front seat would be much more serious than those sitting in back seat if a crash were to occur.

The parents in the CSS user group also had good knowledge regarding the risk of traffic injuries if a child was not properly restrained in the car. When asking parents about reasons why they used or did not use a CSS, the main reason stated by parents in the CSS was that CSS could help secure the child in a car and protect him/her from crash-related injury as well as prevent the child from moving in the car when they drove with the child unaccompanied.

The traffic condition is worrying in the city, I can have my own speed controlled but I can’t control other drivers. Many people don’t drive defensively. It is dangerous. This is why I have to buy a CSS to protect my child.

— mother C in CSS group

Most parents in the non-user group believed that having another adult sitting in the car next to the child was safer than having a child restrained in CSS, and it was unnecessary to have CSS if the adult was present. The biggest concern of these parents was that their child would reject sitting in a CSS. One young mother said she clearly knew that having the child in a CSS is a safe practice, but she had to concede because her child refused to be seated in the CSS, and her mother insisted that holding the baby is the safest way. Though she had a CSS, she had never used it. Another mother in the non-user group shared the similar view and thought CSS was not practical.

I heard that children don’t like to sit in the CSS, even if they do, the time they can use CSS is not long. So it is impractical. It is a waste even if I buy it.

— mother B in CSS non-use group

*2. Having effective parental strategies could help make a difference in child safety restraint use*

Parents of CSS users or non-users reported the barriers of having their children sitting in the CSS, including if a child preferred to sit in the front seat, where he or she can enjoy the better view as the car is moving, and if a child disliked to be restrained in the fixed seat with a belt fastened, which limited their activity. Parents in non-user group easily compromised to the children’s preferences. Parents in the CSS non-use group did place their children sit in the rear of the car. Two mothers in this group required their child to wear a seat belt in the backseat. The rest indicated that they did not bother to have any restraint as long as the child was sitting in the back.

We don’t ask my child to wear the seat belt because most of the time we are driving in the downtown area. My boy sometimes wears it just for fun and then unfastens it in a minute because he feels uncomfortable with that. He isn’t used to it.

— mother D in CSS non-use group

Only one mother in the CSS non-use group would strictly require her child to continue wearing the seatbelt while the car was operating.

I would look in the rearview mirror time to time when driving. If I found my girl unfastened the seat belt, I would remind her or sometimes stop the car on the roadside until she wore it again. It is troublesome.

— mother C in CSS non-use group

Difference was found in parents’ attitudes and practices between the CSS user group and CSS non-user group. Parents in the CSS user group made an effort to train their children to sit in the CSS until a habit was formed. One parent found that using a cartoon card was a very effective way to persuade her daughter to sit in the CSS.

We have cartoon card on a safe travelling education story; my girl loves the story. Once she was unwilling to sit on the CSS, I would remind her by saying “Mika (a character in the story) sits in the CSS, Mika wears the seat belt. What should you do?” She knows the story and would like to behave as Mika does.

— mother C in CSS group

Another parent suggested that linking the CSS to things the child likes could help with developing safe habits.

At the beginning, he didn’t like CSS at all. I tried to convince him by saying that if he sits in the CSS, he could go anywhere or get a candy. He was happy with CSS because there was always incentive associated with his CSS use. As time went by, he was fine with CSS even without any incentive.

— mother D in CSS group

But even in the CSS user group, sometimes, parents compromised to their child’s preference. Two mothers in this group said that they ultimately agreed to let their children not sit in the CSS in part because none of their friends or colleagues used CSS. Their children actually experienced ridicule by their peers, with peers saying things like “only a little baby needs to sit in such a seat” or “you are never growing-up.” Another mother in this group said that “My girl was upset with being sit in CSS and kept asking why she has to sit in it while other girls in her age did not. So I began to tell myself that, ok, she is already five, she can sit well in the backseat now.”

*3. Inadequate promotion and parents’ poor safety awareness contribute to the low rate of child safety restraint in China*

None of the CSS user group used the basket-style infant car seat when their children were infants. All of the parents in CSS user group stated that their children started to sit in the CSS at around 1 year of age, with the earliest user at 9 months. All of the CSSs used were made in China, with price of 1000 *Yuan* or less **(equivalent to $166)**. They had not encountered any quality problems while using these CSSs. Most parents knew about the existence of CSS before their babies were born and tired to buy CSS when they thought it was a right time for their baby.

Lack of publicity of CSS and poor safety awareness on child restraint were listed as the most important reasons for low rate of CSS use in emerging motorization China. For example, CSSs were not seen in any car dealerships. Another reason indicated by parents for the low rate of CSS use was related to general problems with installation and removal. Parents indicated that installing a CSS in a car would take away a seat that could have been available for an adult passenger to use.

No one knows it, how can we use it. I heard it and want to buy it, but I fail to persuade my husband because I don’t know much about it. He said, “Why bother to buy a seat when there are many seats in car?” So I think if it can be sold in the car dealer shop, the salesperson would introduce CSS to their customers.

— mother A in CSS non-use group

When asking about the cost of CSS, most parents expressed they could afford it if it was necessary. Most preferred a rental scheme as CSS may not be used for a long time. When inquiring about their thoughts regarding the effective promotion method(s) to increase the frequency of CSS use, almost all of the parents in the CSS user group suggested that media should publicize CSS, accompanied with stories detailing the huge difference CSS could make during a crash when a child is secured by CSS.

Parents have wrongly believed that holding the children in arm is protective, they have no idea what injury can happen to their baby when in a sudden crash. They should look at the video on vehicle collision, it’s horrible.

I think young parents would like to buy it for their child, but treat it just like a piece of clothes or toy, use it for a while and place it away when their baby doesn’t like it. They themselves don’t know how important the CSS is to their children and they do not have enough patience to teach their kids. It is the parent giving up first. Price is not the obstacle for using.

— mothers in CSS group

*4. Mandatory legislation on child safety restraint use could be an effective approach*

All the parents affirmed their agreement and approval with mandatory legislation on child safety restraint use in the future in China. They thought the most effective way to implement this policy would be to incorporate a fine or deduct points in the current driver’s license point system for non-use. Most parents reported that they knew the Chinese law forbids children to sit in the front seat, but were not sure at what age. They also knew that in other countries, especially western countries, CSS use is mandatory.

How the law can be effectively enforced depends on the executive department. People may not act unless severe punishment is in place, just like current strict punishment on drunk driving or traffic light violations. A fine is needed in the current Chinese society.

— mother E in CSS group

I can accept that, it is a trend, but we need a long-term process. To better implement the regulation, punishment should be effected to fine violators. However, this is not solely the responsibility for the traffic department; educational institutions should also be involved.

— father E in non-use group

### Other thoughts on child passenger safety

Although the purpose of this study was to describe experiences and perceptions on the use of child safety seats, some parents also shared their concerns or life stories on other child passenger safety issues. For example, one participant said she often saw young children get in the car themselves, leaving the backdoor unclosed or unlocked, when their parents picked them up, and there was a great risk for the children to be thrown out when the car was turning. Another mother said it is very dangerous when a small child stands with their head out of the skylight as the car is moving. Some parents mentioned to not leave a child alone in the car, to avoid the child being killed if the car was stolen.

## Discussion

Our previous study using field observation found that while the majority of parent drivers had positive attitudes towards CSS, the rate of usage was extremely low [8]. This in-depth interview provides parents’ experiences and insights on CSS from both users and non-users’ perspectives. The findings showed that misperceptions of motor vehicle hazards and lack of parental patience to teach or enforce their child’s use of CSS were major reasons of not using CSS. Our findings call for both national campaigns on protecting children in a car and media attention covering child occupant safety.

Children are arguably the most precious cargo in a motor vehicle. With the one child policy still prevailing in Chinese society and the high rates of road traffic deaths, it would follow that parents would be eager to have their child optimally protected by safety equipment. Unfortunately, this is not the case. Having the children sitting, accompanied with an adult next to them (either in the rear or in the CSS) was regarded as the safest and ideal driving practice by Chinese parents. Limited knowledge of safe riding may lead parents to the false perception that their young children are “safe enough” in the arms, on the lap of an adult, or just sitting next to him/her [7]. In this study, most interviewed parents were in their 30s with college or above education. However, some of them still felt everything in the rear could be in the control by another adult while at the wheel, or wrongly believed that holding children on an adult’s lap is a better way to protect infants in vehicles [13], and that CSS is the second choice only when the accompanied adult is absent. Actually, these practices cannot protect a baby during a car crash. During rollover crashes, ejection increases an occupant’s risk of severe to fatal injury as compared to risks for those retained in the vehicle [14]. Thus, parents should be taught about the serious threat to their children’s lives if these children are not correctly secured in safety seats. Children could be better protected with optimal restraints and seating during travel.

Studies have showed that when installed and used correctly, CSS reduce the risk of injury by an estimated 71% for infants and 54% for toddlers [15]. Our interview data showed one of the reasons for not installing a CSS was due to parents’ low perceived risk. Many parents did not realize that some of the current practices (including not installing CSS, holding a child on an adult lap, or using seat belt with young child) were unsafe for their child in a car. Future intervention messages should advocate child safety restraint use and correct safe practices with traveling children in cars. Cost was discussed but not found to be a potential barrier for using CSS in this study. However, cost has been raised in other qualitative studies as a barrier to optimal restraint [16]. CSS rental schemes could be acceptable for families with pre-school children. Other options for assisting with costs of optimally restraining children need to be explored. Research has demonstrated that subsidized restraints can assist in increasing appropriate restraint use [17], which may present one solution.

Risk communication research and health behavior theories demonstrate that recognition of personal vulnerability to a hazard is a necessary prerequisite to health behavior change [18,19]. For parents in CSS user groups, their relatively high risk perception of traffic injury urged them to have their children use and continue to use the CSS as long as they can. While for the non-user group, low risk perception and being naïve to susceptibility for motor vehicle injury could be reasons for not using CSS. These parents saw no need to buy a seat when the car does not travel fast or leave downtown. They believed the convenience outweighs the risks in motor vehicle travel. Thus, to increase parent drivers’ perceptions of risk for motor vehicle injury to their children it requires injury prevention messages to have a shocking impact on most parents. If a mother sees that an unrestrained 3-year-old child would have more severe injury than a restrained child during a crash, she may consider using a restraint for her own child. Mass media campaigns are strongly needed and should be specifically tailored for different audiences. Also, the car seat manufacturers and stores should provide alarming safety information when they advertise their products and the media messages should explain the benefits of CSS to the public in the current motorization situation.

A lack of awareness of CSS, low risk perception, and no current mandatory requirement for child safety restraint in China were identified as the leading reasons for lack of child restraint use. For parents in both groups, all indicated that mandatory legislation on child safety restraint use could be an effective approach to increase CSS use. This is due to recent mandatory safe driving behaviors that have been strictly implemented nationally in China. Shantou, for instance, can issue a fine of 200 *Yuan (****$****30)* for driving without a seatbelt. While using social marketing and health education approaches could help increase child safety restraint use as it did with seat belt [20], studies have shown that using laws to regulate child passenger safety are among the most effective mechanisms for decreasing childhood crash injuries among the masses [21,22]. Law enforcement is a more aggressive effective step, with many successful law interventions already being used in more developed countries [23,24]. However, because CSS is less widely available in China and people’s traffic risk perception is low, laws must be well-publicized, comprehensive, and understood [25] as there is still low awareness of child safety seats in China.

### Limitations

This study has several limitations. First, there was a potential selection bias. The sample pool for this study was the participants who had responded to a previous survey on child safety restraint and indicated willingness to be contacted. We contacted parent participants conveniently and thus, the perceptions and experiences that parent participants had might be different from those who had not participated in the study. In addition, the saturation that was reached based on relatively small sample might limit the heterogeneity of the sample and decrease the generalizability of the results. The practical value perhaps could be generalized only to the family that has children under 6 years of age.

## Conclusion

This study documented parents’ perceptions and experiences using CSS. Participating parents felt that the most effective approach to achieve widespread CSS use in China is to combine adequate driving safety education with CSS promotion before legislative interventions are implemented.

## Competing interests

The authors declare that they have no competing interests.

## Authors’ contributions

XC conducted the interview, and drafted the initial manuscript. JY collaborated with LL discussed and identified the key themes, and critically revised the manuscript. CP participated in the design and helped to draft the manuscript. LL designed and supervised the study implementation. All authors read and approved the final manuscript.

## Pre-publication history

The pre-publication history for this paper can be accessed here:

http://www.biomedcentral.com/1471-2458/14/318/prepub
